# A new species of *Goniothalamus* (Annonaceae) from Palawan, and a new nomenclatural combination in the genus from Fiji

**DOI:** 10.3897/phytokeys.32.6663

**Published:** 2013-12-18

**Authors:** Chin Cheung Tang, Bine Xue, Richard M.K. Saunders

**Affiliations:** 1 School of Biological Sciences, The University of Hong Kong, Pokfulam Road, Hong Kong, P. R. China

**Keywords:** *Goniothalamus*, *Polyalthia*, Fiji, Malesia, Melanesia, Palawan, new combination, new species

## Abstract

A new species, *Goniothalamus palawanensis* C.C.Tang & R.M.K.Saunders, **sp. nov.** (Annonaceae), is described from Palawan, Philippines. *Goniothalamus palawanensis* is most closely related to *Goniothalamus amuyon* (Blanco) Merr., but differs in its shorter inner petals, hairy ovaries, and funnel-shaped stigmas. A new nomenclatural combination, *Goniothalamus angustifolius* (A.C.Sm.) B.Xue & R.M.K.Saunders, **comb. nov.**, is furthermore validated to reflect the phylogenetic affinities of a Fijian species previously assigned to *Polyalthia*.

## Introduction

The Annonaceae are a species-rich early-divergent angiosperm family, consisting of ca. 108 genera and ca. 2500 species of trees, scandent shrubs and woody climbers (Chatrou et al. 2012), forming an important component of tropical lowland forest ecosystems. The genus *Goniothalamus* (Blume) Hook. f. & Thomson (subfam. Annonoideae Raf., tribe Annoneae Endl.) is one of the largest genera in the family, with more than 130 species (Nakkuntod et al. 2009). The genus is widely distributed in lowland and submontane forests of tropical South-east Asia, with a centre of diversity in western Malesia, particularly Borneo (34 species: Mat-Salleh 2001; Turner and Saunders 2008), Sumatra (14 species: Saunders 2002) and Peninsular Malaysia/Thailand, south of the Isthmus of Kra (22 species: Saunders 2003; Saunders and Chalermglin 2008).

*Goniothalamus* species are small to large trees, with generally solitary, axillary and pendent inflorescences, and are often cauliflorous or ramiflorous. Individual flowers possess one whorl of three sepals, and two whorls of three petals each, with the outer petals larger than the inner. The three inner petals form a distinctive mitriform dome over the reproductive organs, with three lateral apertures at the base of the dome allowing access to beetle pollinators (Saunders 2010, 2012). The flowers are hermaphroditic, with numerous free stamens and carpels. The stamens have broad connectives that cover the thecae; these connectives vary in length and are taxonomically important. The carpels are variable in ovary indument and the size and shape of the stigmatic head. The fruits are apocarpous, with “monocarps” (derived from individual carpels after fertilisation) that are either sessile or borne on stipes.

Fieldwork in Palawan has revealed a previously unknown *Goniothalamus* species, which is formally described here as *Goniothalamus palawanensis* C.C.Tang & R.M.K.Saunders. The present research also validates a new nomenclatural combination arising from the transfer to *Goniothalamus* of a Fijian species that was formerly classified in *Polyalthia*.

## New species description

### Goniothalamus palawanensis

C.C.Tang & R.M.K.Saunders sp. nov.

urn:lsid:ipni.org:names:77134790-1

http://species-id.net/wiki/Goniothalamus_palawanensis

Figs 1 , 2

#### Diagnosis.

Similar to *Goniothalamus amuyon* (Blanco) Merr. except with shorter inner petals (11–16 mm), hairy ovaries, and filiform pseudostyles with funnel-shaped stigmas.

#### Type.

**Palawan**: Puerto Princesa, Corrigutor, 31 May 2012, *C.C. Tang TCC10* (holotype: L; isotypes: PNH).

#### Description.

Small trees, to 5 m tall, to 3 cm d.b.h. Young shoots (densely) hairy. Leaf laminas 18–31 cm long, 5.8–11 cm wide, length/width ratio 2.3–3.5, broadly elliptic or oblong elliptic, apex (long) acuminate, base acute, papyraceous to coriaceous, 50–100 μm thick, glabrous both ab- and adaxially; midrib slightly pubescent and very prominent abaxially; secondary veins 8 to 10 pairs per leaf, prominent adaxially; tertiary veins reticulate (sometimes slightly percurrent towards base of leaf), distinct; petioles 8.5–15.5 mm long, 1.5–2.8 mm in diameter, hairy. Flowers axillary, solitary, on young branches, pendent; pedicels 8–13(–16.5) mm long, 0.8–1.2(–1.7) mm in diameter, (sparsely) hairy; bracts 2 to 5. Sepals 3–4(–5) mm long, 3.5–4.5(–6.5) mm wide, length/width ratio 0.6–0.9, generally not reflexed at anthesis, not connate, triangular, 170–250 μm thick, (sparsely) hairy abaxially, glabrous to very sparsely hairy adaxially, green, venation indistinct. Outer petals 20.5–34 mm long, 5.5–13.5 mm wide, length/width ratio 2.4–4.9, broadly to elongated lanceolate, 450–1100 μm thick, (densely) hairy both ab- and adaxially, with glabrous region at base of adaxial surface, greenish yellow, venation indistinct. Inner petals 11–16.5 mm long, 5–9.5 mm wide, length/width ratio 1.6–2.5, with 2.3–3.4 mm wide basal claw, 330–800 μm thick, densely hairy abaxially, sparsely hairy adaxially, greenish yellow; apertures between inner petals 3.5–4.5 mm long, 3.5–5 mm wide. Stamens ca. 100 per flower, 1.9–2.2 mm long, 0.3–0.5 mm wide; connectives rounded, 0.2–0.5 mm long, papillate-hairy. Carpels 10 to 15 per flower; ovary 0.8–1.8 mm long, 0.4–0.7 mm wide, densely hairy with long golden-brown hairs; stigmas and pseudostyles 2.4–4 mm long; pseudostyles 0.1–0.3 mm wide, glabrous; stigma funnel-shaped, glabrous. Fruits unknown.

**Figure 1.**
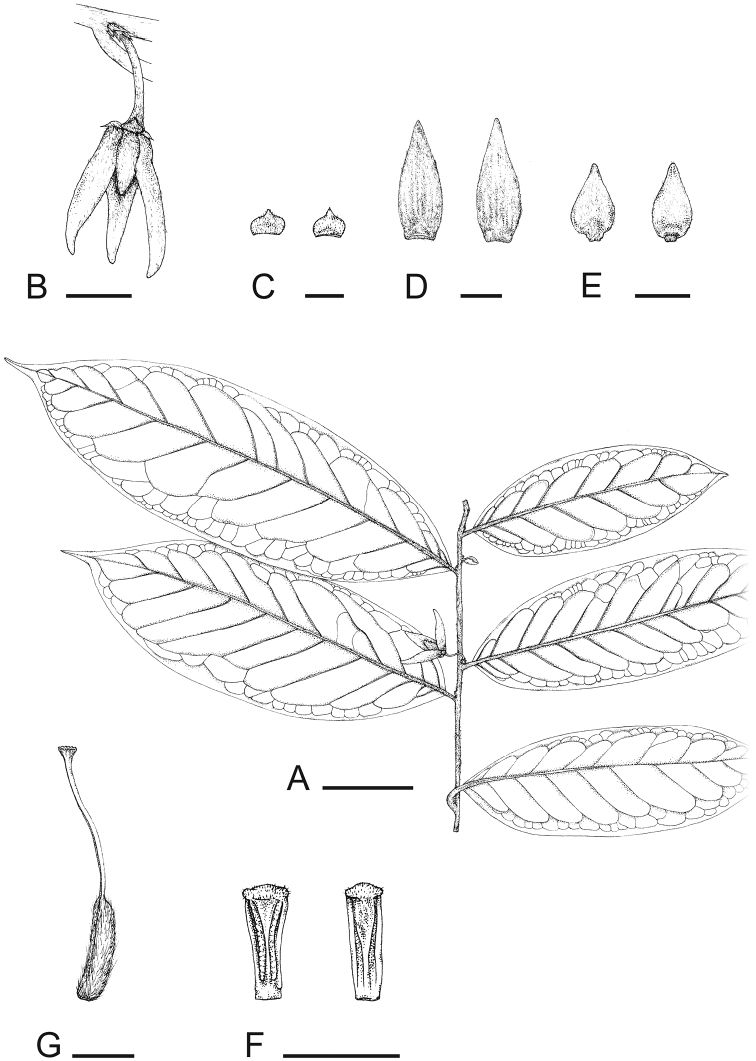
*Goniothalamus palawanensis*, sp. nov.**A** Flowering branch **B** Flower **C** Sepal (ab and adaxial) **D** Outer petal (ab- and adaxial) **E** Inner petal (ab- and adaxial) **F** Stamen (ab- and adaxial) **G** Carpel. Scale bars: **A** = 5 cm; **B, D, E** = 1 cm; **C** = 5 mm; **F** = 2 mm, **G** = 1 mm; **A** from *C.C. Tang 10* (HKU); **B–G** from *C.C. Tang 14* (HKU); drawing by Caren Pearl Shin.

**Figure 2.**
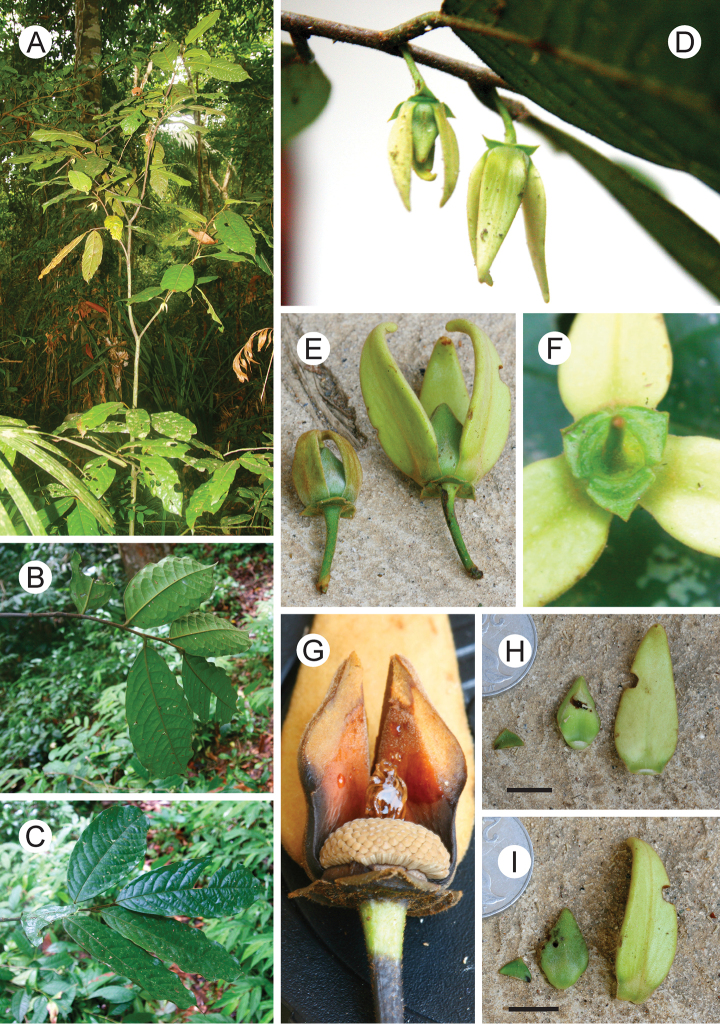
*Goniothalamus palawanensis*, sp. nov. **A** Habit (mature individual with flowers) **B** Branch with leaves (abaxial) **C** Branch with leaves (adaxial) **D, E** Flower **F** Sepals (abaxial) **G** Very mature flower with two outer petals and one inner petal removed, showing stamens and stigmas **H** Perianth parts (abaxial; left to right: sepal, inner petal, outer petal) **I** Perianth parts (adaxial; left to right: sepal, inner petal, outer petal). Scale bars: **H, I** = 1 cm; **A, D** from *C.C. Tang 09* (HKU); **B, C, F, G** from *C.C. Tang 06* (HKU); **E, H**, **I** from *C.C. Tang 14* (HKU). Photos by C.C. Tang.

#### Phenology.

Flowering specimens collected in May and June; fruiting specimens unknown.

#### Distribution and habitat.

**Figure 3.**
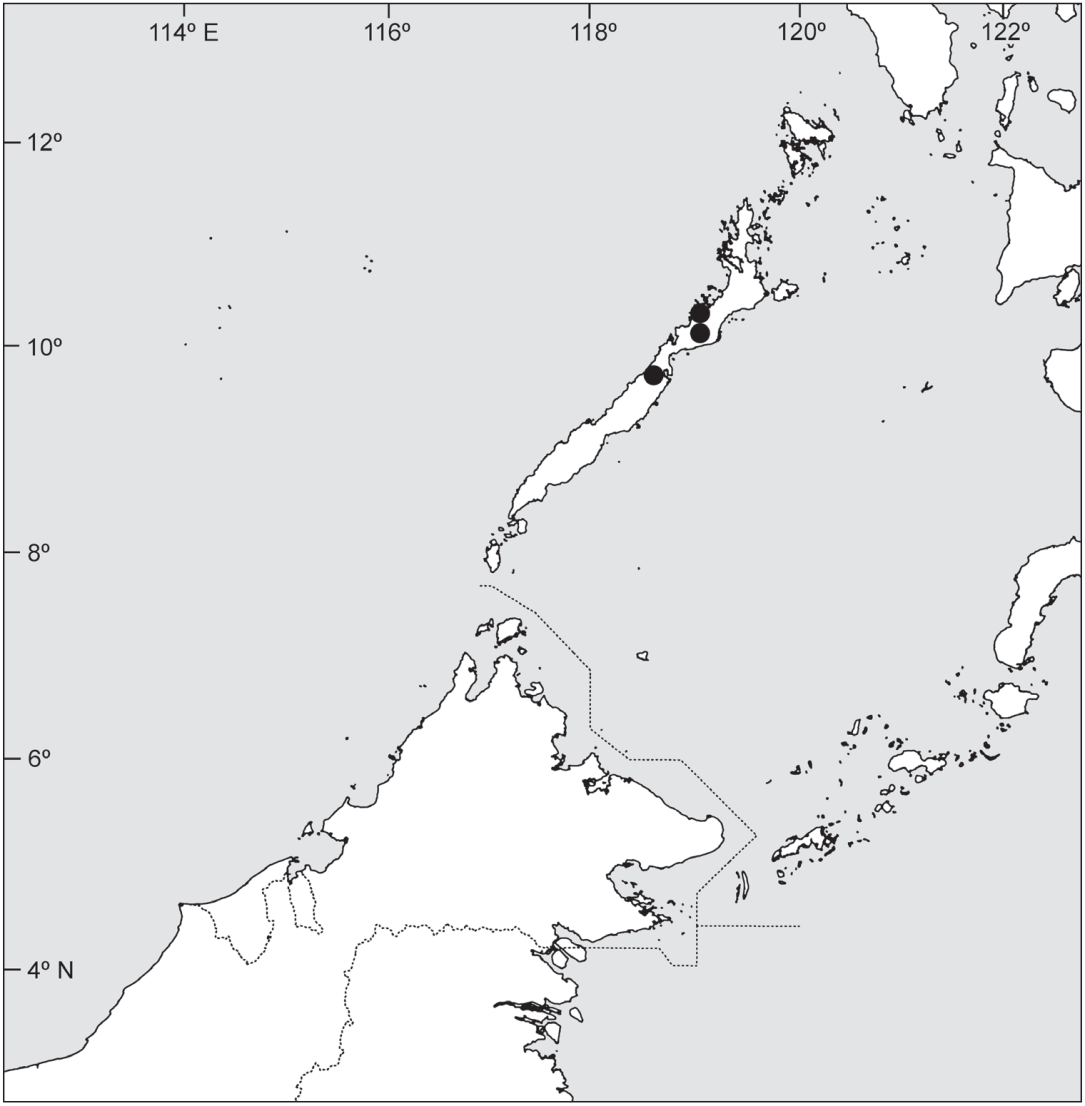
Distribution of *Goniothalamus palawanensis*, sp. nov., in Palawan.

Endemic to Palawan (Fig. 3), in mixed dipterocarp and limestone forests; 50–120 m.

#### Etymology.

The specific epithet reflects the geographical distribution of the species in Palawan.

#### Additional specimens examined (paratypes).

Philippines. Palawan: Bloomfield, St. Pauls Bay, Mt. Bloomfield, lowlands to the SSE, 4 May 1984, *A. C. Podzorski SMHI2012* (K, L); Iraan Mountains, Aborlan, 29 May 1950, *M. D. Sulit 14792* (L); Puerto Princesa, Corrigutor, 31 May 2012, *C.C. Tang TCC06* (HKU), *C.C. Tang TCC09* (HKU), *C.C. Tang TCC11* (HKU), *C.C. Tang TCC14* (HKU), *C.C. Tang TCC17* (HKU).

#### Discussion.

Phylogenetic analysis of chloroplast DNA sequence data (C.C. Tang et al., unpubl.) indicates that this new species, *Goniothalamus palawanensis*, is sister to *Goniothalamus amuyon* (Blanco) Merr. with moderate to strong support (posterior clade probability = 0.97 and bootstrap support = 74%), and more distantly related to *Goniothalamus costulatus* Miq., *Goniothalamus rufus* Miq., *Goniothalamus sawtehii* C.E.C.Fischer, *Goniothalamus tomentosus* R.M.K.Saunders, *Goniothalamus undulatus* Ridl. and *Goniothalamus velutinus* Airy-Shaw. These species are all characterised by a distinct indument of rusty-red hairs on the young shoots and petals. Amongst these species, *Goniothalamus amuyon* and *Goniothalamus palawanensis* are distinct in possessing fewer secondary veins per leaf (8 to 11, compared with 11 to 25 in the other species, with the exception of *Goniothalamus rufus*), and in having indistinct sepal venation (although similar venation is observed in *Goniothalamus velutinus*). *Goniothalamus palawanensis* is furthermore geographically close to *Goniothalamus amuyon*, which occurs in Luzon, Visayas and Mindanao (Guzman et al. 1986). Morphological differences between *Goniothalamus palawanensis* and *Goniothalamus amuyon* include: inner petal length (11–16.5 mm vs 15–29 mm, respectively: Ying 1991; Liao 1996); ovary indument (hairy in *Goniothalamus palawanensis* [Fig. 1G] vs glabrous in *Goniothalamus amuyon*); and pseudostyle/stigma shape (filiform pseudostyle with small, funnel-shaped stigma in *Goniothalamus palawanensis* [Fig. 1G], vs relatively enlarged, fleshy pseudostyle with entire stigma in *Goniothalamus amuyon*).

The flora of Palawan shows close biogeographical affinities with Borneo, reflecting the extensive connectivity that existed between the two regions (Hall 2009). Two of the species listed above as close relatives of *Goniothalamus palawanensis* occur in Borneo, viz. *Goniothalamus rufus* and *Goniothalamus velutinus*. In addition to the differences in leaf and sepal venation alluded to above, these species differ from *Goniothalamus palawanensis* in possessing greatly enlarged and warty pseudostyles/stigmas (Mat-Salleh 1993).

There is only one *Goniothalamus* species, *Goniothalamus obtusifolius* Merr., that is sympatric with *Goniothalamus palawanensis* in Palawan. These two species are clearly distinct, however, as *Goniothalamus obtusifolius* has much smaller (15–18 × 6–8 cm) coriaceous leaves, and large (ca. 5 × 3.5 cm) membranous outer petals (Merrill 1906).

#### IUCN conservation status.

EN B1ab(iii) (IUCN, 2001). *Goniothalamus palawanensis* is endemic to Palawan, with an extent of occurrence of ca. 1,800 km^2^. The species is only known from three periods of collection (1950, 1984 and 2012), and from fewer than five localities. The region is subject to continuing habitat decline due to logging of low altitude forests (DENR/UNEP 1997), hence the endangered red list category recommendation.

## New nomenclatural combination

### Goniothalamus angustifolius

(A.C.Sm.) B. Xue & R.M.K.Saunders comb. nov.

urn:lsid:ipni.org:names:77134791-1

http://species-id.net/wiki/Goniothalamus_angustifolius

#### Basionym.

*Polyalthia angustifolia* A.C.Sm., Bull. Torrey Bot. Club 70: 538. 1943. Type: FIJI: Viti Levu, *J.W. Gillespie 2198* (holotype: A!; isotypes: BISH, GH!).

#### Discussion.

The historical delimitation of the genus *Polyalthia* has been shown to be highly polyphyletic, and large-scale taxonomic realignment and recognition of new genera has been undertaken to ensure strict monophyly of genera (Mols et al. 2008; Saunders et al. 2011; Xue et al. 2011, 2012, in press; Chaowasku et al. 2012). As part of this series of taxonomic revisions, chloroplast DNA regions were sequenced from eight species from the Melanesian island of Fiji (Xue, 2013) that had previously been assigned to *Polyalthia*. Phylogenetic analysis of this data revealed that most of these species align with either *Hubera* (Chaowasku et al. 2012; Xue 2013) or *Meiogyne* (Xue 2013; Xue et al. in press), although one species, *Polyalthia angustifolia* A.C.Sm., which was sequenced from the type material, is nested within the *Goniothalamus* clade (Xue 2013). *Polyalthia angustifolia* was originally described from fruiting material (Smith 1943), and it is likely that its incorrect generic affiliation was due to the absence of flowers, which are very different in *Polyalthia* and *Goniothalamus*. Subsequent phylogenetic analyses with a larger taxon sampling (C.C. Tang et al., unpubl.) have revealed *Polyalthia angustifolia* as sister to the Fijian species *Goniothalamus monospermus* (Baill.) R.M.K.Saunders with strong support (posterior clade probability = 1; bootstrap support = 96%); these two species are morphologically distinct, as *Polyalthia angustifolia* seeds lack the broad lateral testa wings that are diagnostic of *Goniothalamus monospermus* (Van Setten and Koek-Noorman 1992: pl. 39i). The transfer of the name *Polyalthia angustifolia* to *Goniothalamus* is accordingly validated here.

## Supplementary Material

XML Treatment for Goniothalamus palawanensis

XML Treatment for Goniothalamus angustifolius
